# Favipiravir treatment prolongs the survival in a lethal mouse model intracerebrally inoculated with Jamestown Canyon virus

**DOI:** 10.1371/journal.pntd.0009553

**Published:** 2021-07-02

**Authors:** Hirofumi Kato, Mutsuyo Takayama-Ito, Masaaki Satoh, Madoka Kawahara, Satoshi Kitaura, Tomoki Yoshikawa, Shuetsu Fukushi, Nozomi Nakajima, Takashi Komeno, Yousuke Furuta, Masayuki Saijo

**Affiliations:** 1 Department of Virology I, National Institute of Infectious Diseases, Tokyo, Japan; 2 Department of Internal Medicine, The University of Tokyo, Graduate School of Medicine, Tokyo, Japan; 3 FUJIFILM Toyama Chemical Co., Ltd., Toyama, Japan; Australian Red Cross Lifelood, AUSTRALIA

## Abstract

**Background:**

Jamestown Canyon virus (JCV) is a mosquito-borne orthobunyavirus that causes acute febrile illness, meningitis, and meningoencephalitis, primarily in North American adults. Currently, there are no available vaccines or specific treatments against JCV infections.

**Methodology/Principal findings:**

The antiviral efficacy of favipiravir (FPV) against JCV infection was evaluated *in vitro* and *in vivo* in comparison with that of ribavirin (RBV) and 2’-fluoro-2’-deoxycytidine (2’-FdC). The *in vitro* inhibitory effect of these drugs on JCV replication was evaluated in Vero and Neuro-2a (N2A) cells. The efficacy of FPV in the treatment of JCV infection *in vivo* was evaluated in C57BL/6J mice inoculated intracerebrally with JCV, as per the survival, viral titers in the brain, and viral RNA load in the blood. The 90% inhibitory concentrations (IC_90_) of FPV, RBV, and 2’-FdC were 41.0, 61.8, and 13.6 μM in Vero cells and 20.7, 25.8, and 8.8 μM in N2A cells, respectively. All mice infected with 1.0×10^4^ TCID_50_ died or were sacrificed within 10 days post-infection (dpi) without treatment. However, mice treated with FPV for 5 days [initiated either 2 days prior to infection (−2 dpi–2 dpi) or on the day of infection (0 dpi–4 dpi)] survived significantly longer than control mice, administered with PBS (p = 0.025 and 0.011, respectively). Moreover, at 1 and 3 dpi, the virus titers in the brain were significantly lower in FPV-treated mice (0 dpi–4 dpi) *versus* PBS-treated mice (p = 0.002 for both 1 and 3 dpi).

**Conclusions/Significance:**

Although the intracerebral inoculation route is thought to be a challenging way to evaluate drug efficacy, FPV inhibits the *in vitro* replication of JCV and prolongs the survival of mice intracerebrally inoculated with JCV. These results will enable the development of a specific antiviral treatment against JCV infections and establishment of an effective animal model.

## Author summary

Jamestown Canyon virus (JCV) is a mosquito-borne virus (arbovirus) classified into the California serogroup. JCV is distributed widely throughout North America and is considered one of the potentially re-emerging viruses due to the recent spurt in JCV cases in the region. JCV infection often leads to an acute febrile illness, meningitis, and meningoencephalitis mainly among adults. Currently, no antiviral therapy against JCV is approved. In this study, we evaluated the antiviral efficacy of favipiravir (FPV), ribavirin (RBV), and 2’-fluoro-2’-deoxycytidine (2’-FdC) against JCV infection in cultured cells and mice. As a result, FPV, RBV, and 2’-FdC effectively inhibited JCV replication in Vero and Neuro-2a cells. Furthermore, FPV delayed the onset of neurological symptoms in mice intracerebrally inoculated with JCV. Notably, although most patients infected with JCV do not present severe disease, neuroinvasive cases are not rare and may result in residual neurological sequelae such as persisting cognitive deficits. Therefore, this study contributes to the development of a specific antiviral treatment for patients with JCV infection.

## Introduction

Jamestown Canyon virus (JCV), a mosquito-borne virus (arbovirus), belongs to the genus *Orthobunyavirus* in the family *Peribunyaviridae* of the order Bunyavirales [1]. JCV is one of the California serogroup (CSG), together with La Crosse virus (LACV), snowshoe hare virus (SSHV), Inkoo virus (INKV), and Tahyna virus (TAHV) [2]. Of note, CSG viruses cause disease in humans all over the world: North America (LACV and SSHV), Asia and Europe (INKV, TAHV), North and South America (Guaroa virus), and Africa (Lumbo virus) [3,4].

JCV was first isolated in 1961 from *Culiseta inornate* mosquitoes at Jamestown Canyon, Colorado, in the United States [5], and is distributed widely throughout North America. Recently, the number of JCV cases has been increasing; in the northern region of the United States, 0–3 JCV cases had been reported by 2012, however, the number of cases increased to 75 and 41 cases in 2017 and 2018, respectively [6–8]. Therefore, JCV is considered as one of the potentially re-emerging viruses, threatening public health. The main vectors of JCV are thought to be mosquitoes, including different species within the genera *Aedes*, *Coquillettidia*, *Culex*, and *Culiseta* [9,10]; the main reservoirs include mammalian hosts such as white-tail deer, mules deer, sika deer, moose, horses, and goats [3,11].

JCV often causes an acute febrile illness, that may evolve to meningitis or meningoencephalitis, mainly among adults [12,13]. Epidemiological studies on human JCV infections demonstrate that the most common symptoms include fever, generalized weakness, headache, myalgia, and nausea; neurological signs such as neck rigidity, altered mental status, disturbance in balance, dizziness, and photophobia were also observed [12,13]. Importantly, the studies reported that approximately 50–80% of patients developed neuroinvasive disease and 50% of patients required admission to the hospital; in fact, 10% required mechanical ventilation, and one out of 30 patients died of the condition [13]. Further more, it has also been reported that after the resolution of the infection, patients who developed neuroinvasive disease may show residual neurological sequelae such as persisting cognitive deficits [7,14].

Therefore, the development of vaccines or treatments against JCV is essential. Although a vaccine candidate against JCV was developed [15], to date there are no approved vaccines and specific treatments for humans. In this study, we focused on three potential antivirals for the treatment of JCV: favipiravir (FPV, 6-fluoro-3-hydroxy-2-pyrazinecarboxamide, also known as T-705), ribavirin (RBV), and 2’-fluoro-2’-deoxycytidine (2’-FdC). FPV is a broad-spectrum inhibitor and has exhibited efficacy in controlling infections associated with various RNA viruses [16], such as influenza viruses [17,18], Crimean-Congo hemorrhagic fever virus (CCHFV) [19], severe fever with thrombocytopenia syndrome virus (SFTSV) [20], West Nile virus [21], Western equine encephalitis virus [22], and rabies virus [23] under *in vitro* and/or *in vivo* conditions. FPV is phosphoribosylated in cells to an active form, which is recognized as a purine nucleoside by the viral RNA-dependent RNA polymerase (RdRp), thereby inhibiting its activity [24]. In a clinical setting, FPV has been approved for use against the emerging pandemic influenza virus in Japan. Additionally, clinical trials have shown that FPV improved survival in patients with Ebola virus disease [25,26] and Coronavirus disease 2019 (COVID-19) [27,28]. RBV is a guanosine analog with broad-spectrum activity against RNA and DNA viruses [29]. RBV showed efficacy, under experimental conditions, against Lassa fever virus [30], Nipah virus [31], Junin virus [32], and Rift Valley fever virus (RVFV) [33]. Lastly, 2’-FdC is a nucleotide analog and a broad-spectrum inhibitor of various RNA viruses including CCHFV [34], SFTSV, and RVFV [35].

Here, we evaluated the antiviral efficacy of these three drugs against JCV infection *in vitro*, using Vero and Neuro-2a (N2A) cells, and *in vivo*, in the context of a lethal mouse model.

## Methods

### Ethics statement

The animal experiments were carried out as previously described [36,37]. All animal studies were performed in strict accordance with the recommendations described in the Guidelines for Proper Conduct of Animal Experiments of the Science Council of Japan, and in strict compliance with the animal husbandry and welfare regulations. All animal experiments were reviewed and approved by the Institutional Animal Care and Use Committee of the National Institute of Infectious Diseases (NIID) (approval Nos. 119084 and 119129). All animals infected with JCV were handled in biosafety level 2 animal facilities in accordance with the NIID guidelines. Mice were inoculated with viruses under anesthesia, as described above.

### Cells and viruses

Vero cells were purchased from the American Type Culture Collection (ATCC, Manassas, VA, USA; #CCL-81). Neuro-2a (N2A) cells were obtained from the JCRB cell bank (IFO50091), originating from the ATCC (#CCL-131). Vero cells were grown in Dulbecco’s modified Eagle medium (DMEM; Sigma-Aldrich, St. Louis, MO, USA) supplemented with 5% heat-inactivated fetal bovine serum (FBS; Biowest, Nuaille, France), non-essential amino acids (Sigma-Aldrich), and antibiotics (100 U/mL penicillin and 100 μg/mL streptomycin; Thermo Fisher Scientific, Waltham, MA, USA). N2A cells were grown in DMEM supplemented with 10% heat-inactivated FBS and antibiotics. Jamestown canyon viruses (JCV; 61V-2235 strain) were purchased from the ATCC (VR-712). Mycoplasma contamination in the virus solution and cell cultures was checked using the CycleavePCR Mycoplasma Detection Kit (TaKaRa Bio Inc., Otsu, Japan) and a LightCycler 96 (Roche Life Science, Penzberg, Germany). No mycoplasma contamination was found in the virus solution and the cell culture.

### Antiviral compounds

FPV was provided by FUJIFILM Toyama Chemical Co., Ltd. (Toyama, Japan). RBV and 2’-FdC were purchased from FUJIFILM Wako Pure Chemical Corporation (Osaka, Japan) and Tokyo Chemical Industry Co., Ltd. (Tokyo, Japan), respectively. All drugs were dissolved in DMEM supplemented with 2% FBS (DMEM-2FBS) or in 10 μL of dimethyl sulfoxide (DMSO) and 90 μL of phosphate-buffered saline (PBS) per mouse for *in vitro* and *in vivo* experiments, respectively.

### Virus titration

The infectious doses of the viruses were determined via a 50% tissue culture infectious dose (TCID_50_) infectivity assay. Briefly, Vero cell monolayers were infected with 10-fold serial dilutions of viral supernatants and cultured at 37°C for 5 days in DMEM-2FBS. After the incubation period, the cells were fixed with 10% formalin for 1 hour and stained with methylene blue solution. After washing with distilled water, the cytopathic effect (CPE) was examined. For each dilution, the number of CPE positive wells was recorded. The Spearman-Karber method was used to determine the 50% infectious dose, which was scaled up to obtain the TCID_50_ per mL.

### Virus yield reduction assay

Vero cells were infected with JCV at a multiplicity of infection (MOI) of 0.001, in the presence of 0, 1, 3, 10, 30, 100, 300, and 1000 μM of FPV, RBV, and 2’-FdC and cultured at 37°C for 2 days, whereas N2A cells were infected at a MOI of 0.1 with identical drug treatments and cultured at 37°C for 3 days. The supernatants were then collected and the virus infectious dose was determined via TCID_50_, as described above.

### Cytotoxicity *in vitro* assay

Cytotoxicity was measured as previously described [38]. Briefly, Vero and N2A cells (non-infected) were cultured for 2 and 3 days, respectively, in the presence of different drugs at the designated concentrations. Cell viability was measured using the cell counting kit-8 (CCK-8; Dojindo, Kumamoto, Japan) according to the manufacturer’s protocol. Cell viability was calculated as follows: [(absorbance (Abs) of cells cultured in the presence of drug—Abs of no cells in the absence of drug) / (Abs of cells in the absence of drug—Abs of no cells in the absence of drug)] x 100.

### Quantitative reverse transcription real-time PCR (qRT-PCR)

Total RNA was prepared from 200 μL of blood samples using the NucleoSpin RNA Blood kit (Macherey-Nagel, Düren, Germany). RNA was subjected to OneStep RT-qPCR analysis using the Luna Universal Probe One-Step RT-qPCR Kit (New England Biolabs, Beverly, MA, USA), with 1 μL template and 10 μM of primers and probe in a 20-μL reaction volume according to the manufacturer’s protocol. Fluorescence signals were measured using a LightCycler 96 (Roche Life Science). The JCV-specific PCR primers and probe were designed within the S segment; the forward and reverse primers used were 5’-TGATGTCGCATCCACAGGTG-3’ and 5’-TCCGGTTTACGAGCGAGAGC-3’, respectively; the ZEN double-quenched probe (Integrated DNA Technologies, Coralville, IA, USA) used was 5´-56-FAM-TGGCTGACC/ZEN/ACGGAGAGTCTATCA-3IABkFQ-3’. The amplification conditions were as follows: 55°C for 10 min, 95°C for 1 min, 45 cycles of 95°C for 10 s, and 60°C for 30 s.

### Development of a JCV infection lethal mouse model

Five-week-old female C57BL/6J mice were purchased from Japan SLC (Shizuoka, Japan) and allowed to acclimate for 1 week. To determine the lethal dose of JCV, mice (n = 6/dose) were inoculated intranasally (2 μL) or intracerebrally (30 μL) with 10-fold serially diluted JCV. All mice were anesthetized with isoflurane before intranasal and intracerebral inoculation. Bodyweight was recorded daily for 2 weeks, and each mouse was observed daily for the development of neurological signs including lethargy, tremors, ataxia, circling, paralysis, and seizures.

### Evaluation of *in vivo* drug efficacy

Five-week-old female C57BL/6J mice were purchased from Japan SLC and allowed to acclimate for 1 week. To evaluate drug efficacy, mice (n = 5/group) were inoculated intracerebrally with a volume of 30 μL containing 1.0×10^4^ TCID_50_ of JCV. Additionally, mice received intraperitoneal administration of the following drugs once a day: FPV (300 mg/kg/day), RBV (100 mg/kg/day), 2’-FdC (100 mg/kg/day), or the same volume of PBS as negative control. Treatments commenced 2 days prior to virus inoculation (pre-treatment group) or on the same day of virus inoculation (simultaneous-treatment group) and then continued for 5 days. Each mouse was observed daily for the development of neurological signs including lethargy, tremors, ataxia, circling, paralysis, and seizures. Survival was also recorded. A total of 2 independent experiments were conducted. The infectious viral titers in the brain and whole blood viral RNA levels were also measured in the context of FPV- and PBS-treated mice. Briefly, brain and whole blood samples were collected 1, 3, and 5 dpi. Animals were subjected to isoflurane anesthesia and were euthanized; transcardial PBS perfusion was performed before tissue collection. All samples were stored at −80°C until further use.

### Virus titration of blood and brain samples

Brain samples of mice (0.5 g) inoculated with JCV were collected and 750 μL of PBS was added to it, followed by homogenization using a TissueLyser II (QIAGEN, Hamburg, Germany). The supernatants were collected after centrifugation (4,000 x *g*, 10 minutes, 4°C) and further diluted to a 10-fold emulsion. Blood samples were collected in DNA LoBind Tubes, followed by the addition of heparin to avoid coagulation, and diluted 100-times with DMEM-2FBS. The virus infectious doses of brain and blood samples were determined on Vero cells via TCID_50_, as described above.

### Statistical analysis

The log-rank test was used to compare the characteristics of the different groups with respect to the Kaplan-Meier curves. The Mann–Whitney U test was used to compare the virus titers in the brain and the viral RNA levels in the blood of the different groups. All p-values were two-tailed, and a p < 0.05 was considered significant. All data were analyzed using GraphPad Prism 8 for Windows (Graphpad Software Inc., San Diego, CA, USA).

### Humane endpoints

Overall, the animal experiments were carried out as previously described [36,37]. In this study, humane endpoints were adapted in line with early indicators of animal pain or distress; of note, such indicators can be used to avoid or limit animal suffering via the adoption of actions such as humane euthanasia. During the observation period, neurological symptoms were monitored daily. The humane endpoint was defined as mice reaching a moribund stage (persistent JCV-associated clinical signs after infection, such as lethargy, tremors, ataxia, circling, paralysis, and seizures). Moribund mice were euthanized under isoflurane anesthesia immediately after they met the endpoint criteria. All of the research staffers were specially trained in animal care and treatment under the standard operation procedures of our laboratory. Two investigators, who were not blinded to the treatment, mainly determined whether mice met the endpoint.

## Results

### *In vitro* antiviral activity against JCV

**Fig 1:**
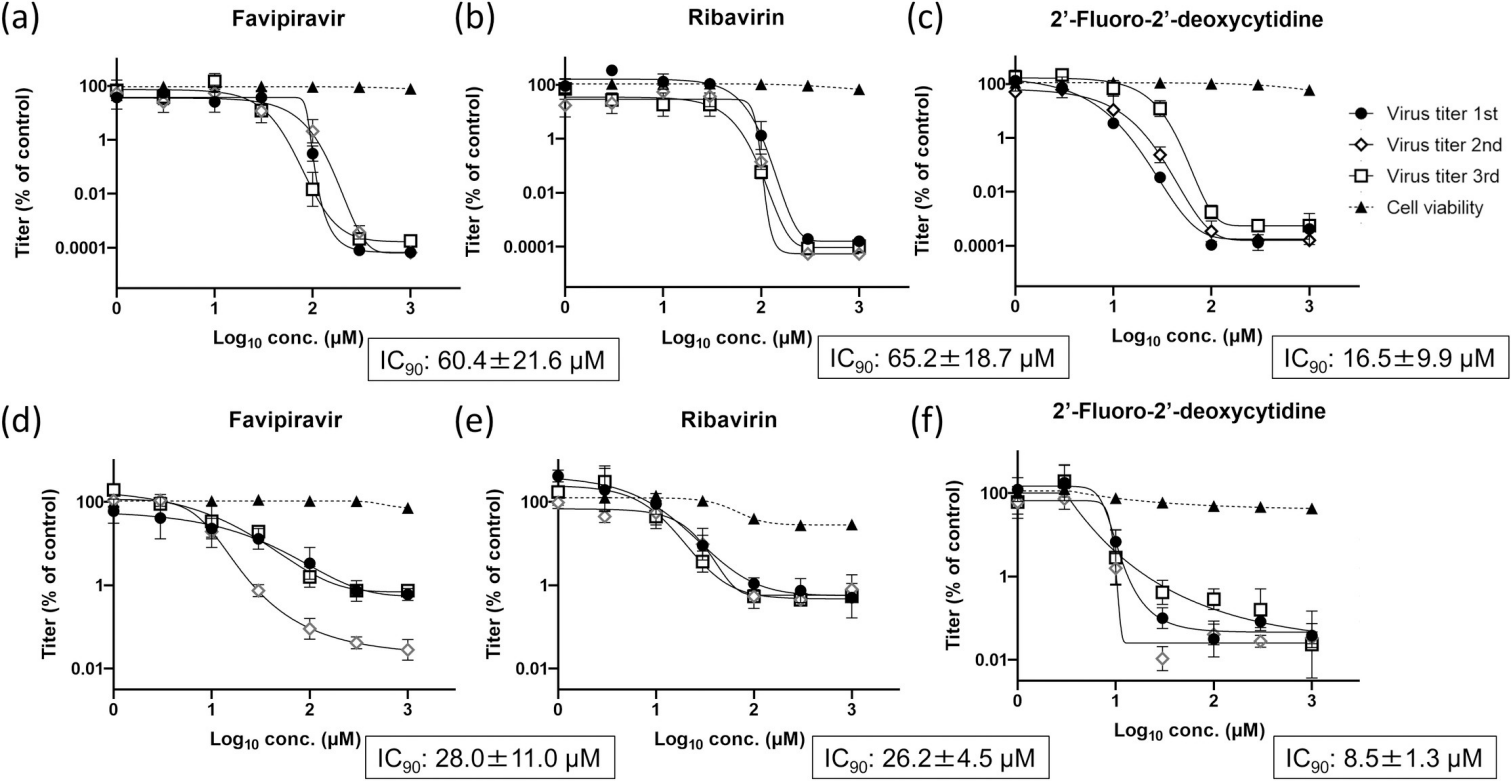
Inhibitory effects of FPV, RBV, and 2’-FdC on the replication of JCV in Vero and N2A cells. The inhibitory effect of FPV (a), RBV (b), and 2’-FdC (c) on the replication of JCV in Vero cells and the respective cytotoxic effect were assessed. The same parameters were measured in the context of N2A cells (d-f). Vero cells and N2A cells were infected with JCV at a multiplicity of infection of 0.001 and 0.1 in the presence of various concentrations of FPV, RBV, and 2’-FdC, cultured at 37°C for 2 days and 3 days, respectively. Cell viability was evaluated in non-infected cells treated under the same conditions of drugs. Three wells were used in a single independent experiment and then three independent experiments were performed. The relative titers were calculated and are shown as mean values with standard deviations.

The antiviral activity of FPV, RBV, and 2’-FdC against JCV was evaluated in Vero and N2A cells (Fig 1). Without the drugs, the titer of JCV in Vero cells and N2A cells reached approximately 1×10^7^ TCID_50_/mL and 1×10^6^ TCID_50_/mL in 2 and 3 days, respectively. The 90% inhibitory concentrations (IC_90_) of FPV, RBV, and 2’-FdC in Vero cells were 60.4±21.6, 65.2±18.7, and 16.5±9.9 μM, respectively. Of note, none of the drugs affected cell viability (considering the relevant concentration range), as per the CCK8 assay. Additionally, the IC_90_ of FPV, RBV, and 2’-FdC in N2A cells were 28.0±11.0, 26.2±4.5, and 8.5±1.3 μM, respectively. FPV did not affect cell viability (except for the 1000-μM dose), and RBV and 2’-FdC were not cytotoxic in the context of concentrations lower than 100 and 30 μM, respectively. As the concentration of the three drugs increased, the virus titers decreased with a fitting mode of a sigmoid curve, indicating that the drugs inhibited the replication of JCV in a dose-responsive manner.

### Development of a JCV infection lethal mouse model

**Fig 2:**
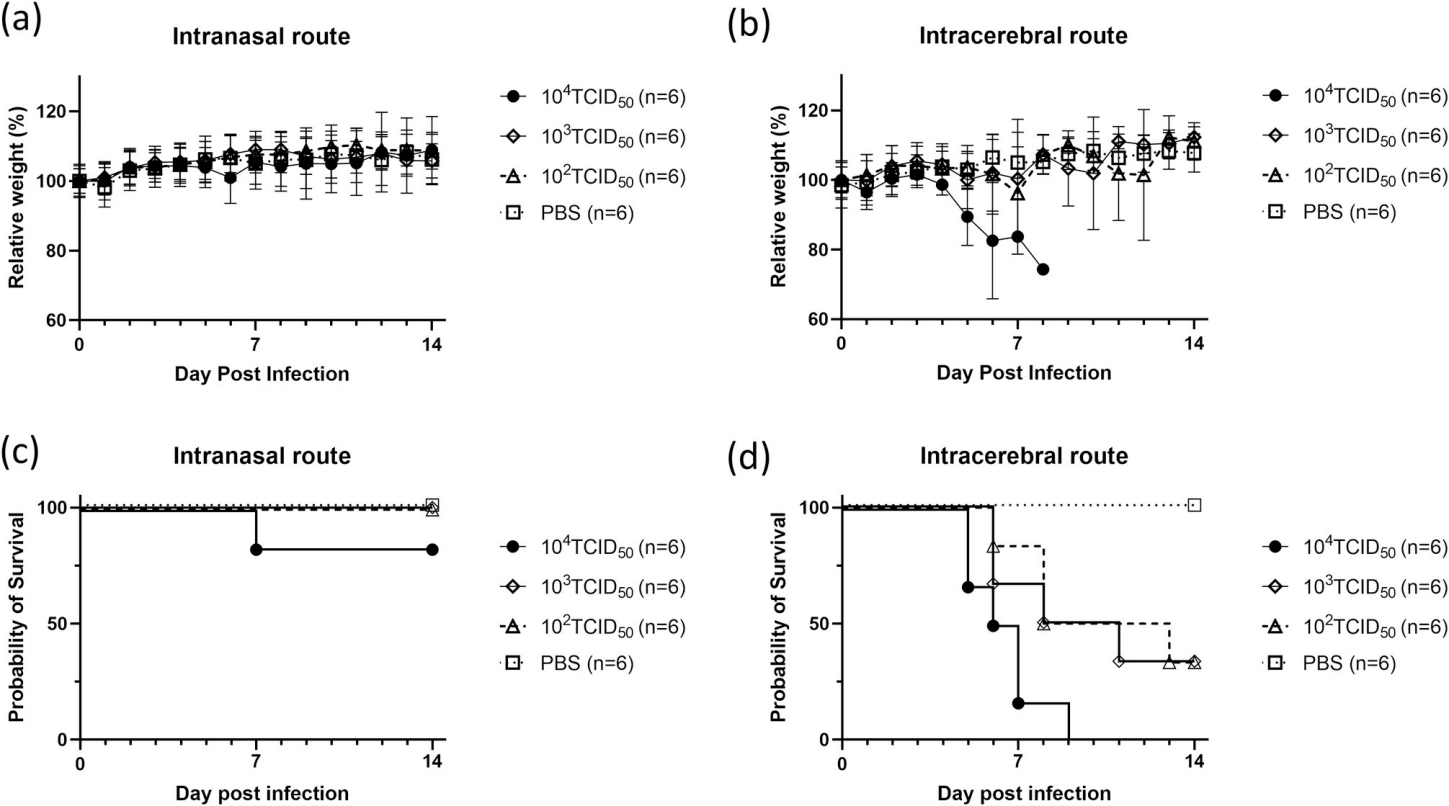
Survival curves and body weight dynamics of C57BL/6J mice infected intracerebrally and intranasally with different JCV titers. Percent body-weight change (a, b) and survival (c, d) of C57BL/6J mice intracerebrally and intranasally inoculated with JCV are shown. Six female mice in each group were inoculated with different doses of JCV and PBS as a control. Survival was determined using Kaplan-Meier analysis. Relative weight was calculated and is shown as mean values with standard deviations. Data were obtained from a single experiment.

To evaluate the efficacy of the three drugs *in vivo*, a JCV infection lethal mouse model was developed in the present study. The changes in bodyweight and survival curves of C57BL/6J mice inoculated intranasally or intracerebrally with various titers of JCV and PBS are shown in Fig 2. In the group inoculated intranasally with 1.0×10^4^ TCID_50_ JCV, one of the six mice (16.7%) died 7-days post-infection (dpi) before meeting the criteria for euthanasia; however, all of the other mice survived without any neurological symptoms and weight loss. Additionally, in the groups inoculated intranasally with 1.0×10^2^ TCID_50_ and 1.0×10^3^ TCID_50_ of JCV, or with PBS, all mice survived without any neurological symptoms and weight loss. In contrast, in the group inoculated intracerebrally with 1.0×10^4^ TCID_50_ JCV, all mice died or were sacrificed within 9 dpi. However, in the groups of mice inoculated intracerebrally with 1.0×10^2^ TCID_50_ and 1.0×10^3^ TCID_50_ JCV, two of the six mice (33.3%) survived during the observation period; of note, all the mice inoculated with PBS survived without any weight reduction.

### *In vivo* efficacy of the antiviral drugs in the JCV mouse model

**Fig 3:**
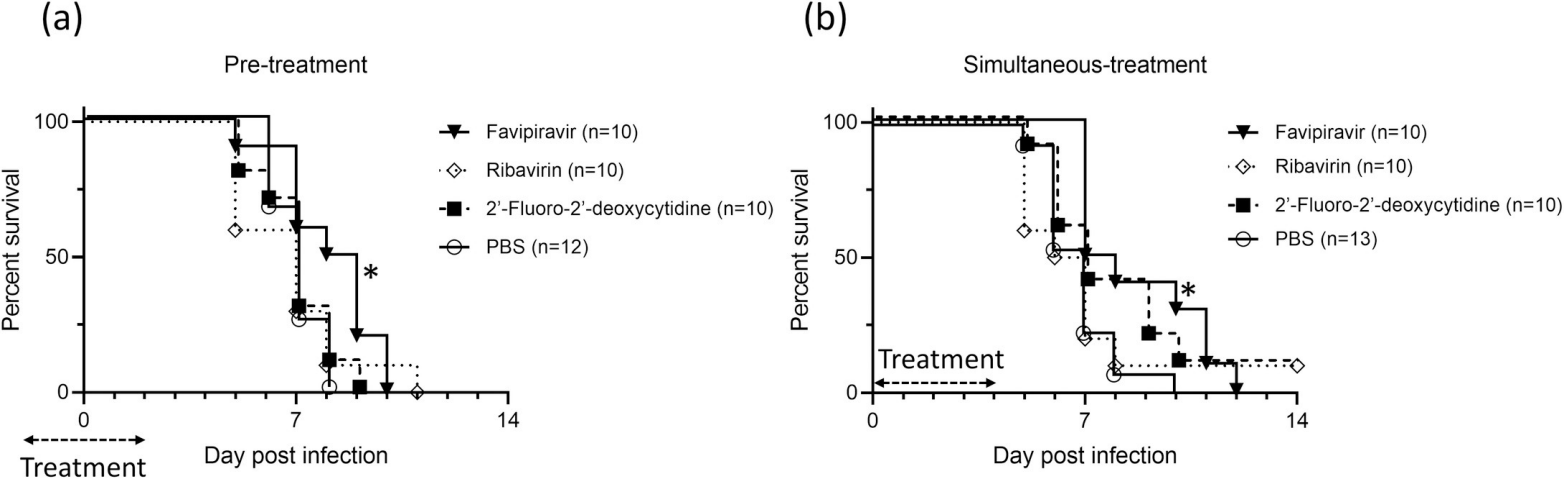
Survival of JCV-infected C57BL/6J mice treated with FPV, RBV, and 2’-FdC. Five mice in each group were inoculated intracerebrally with 1.0×10^4^ TCID_50_ JCV. The combined data of the two independent experiments are shown. Treatments were commenced (a) 2 days prior to virus inoculation (pre-treatment) or (b) on the same day of virus inoculation (simultaneous-treatment) and continued for 5 days. The mice were treated intraperitoneally with FPV (300 mg/kg/day), RBV (100 mg/kg/day), 2’-FdC (100 mg/kg/day), or PBS as negative control once a day. Survival was determined using the Kaplan-Meier analysis. Asterisks denote significant differences (p < 0.05).

The survival curves of JCV-infected C57BL/6J mice treated with FPV, RBV, or 2’-FdC are shown (Figs 3 and S1). The mice treated with PBS (negative control) showed almost the same survival curve as that of the mice inoculated with 1.0×10^4^ TCID_50_ in Fig 2 and died within 8 dpi. In the pre-treatment group, all mice treated with FPV died within 10 dpi; of note, the mice survived significantly longer compared to control mice (p = 0.025). All mice treated with RBV and 2’-FdC died within 11 and 9 dpi, respectively; however, no difference was found between these groups and the control group treated with PBS. In the simultaneous-treatment group, one of the ten mice (10%) treated with RBV and 2’-FdC survived during the observation period, while all mice treated with FPV and PBS died. There was a significant difference in the time from infection to death between the mice treated with FPV and those treated with PBS (p = 0.011). Detailed data of each experiment are shown in S1 Table. No difference was found between those treated with other drugs and the PBS controls. Importantly, while all of the mice treated with FPV started to die from 7 dpi, mice in the remaining groups started to perish from day 5 dpi.

### Comparison of the virus titers in the brain and blood JCV RNA levels in FPV *versus* PBS treated animals

**Fig 4:**
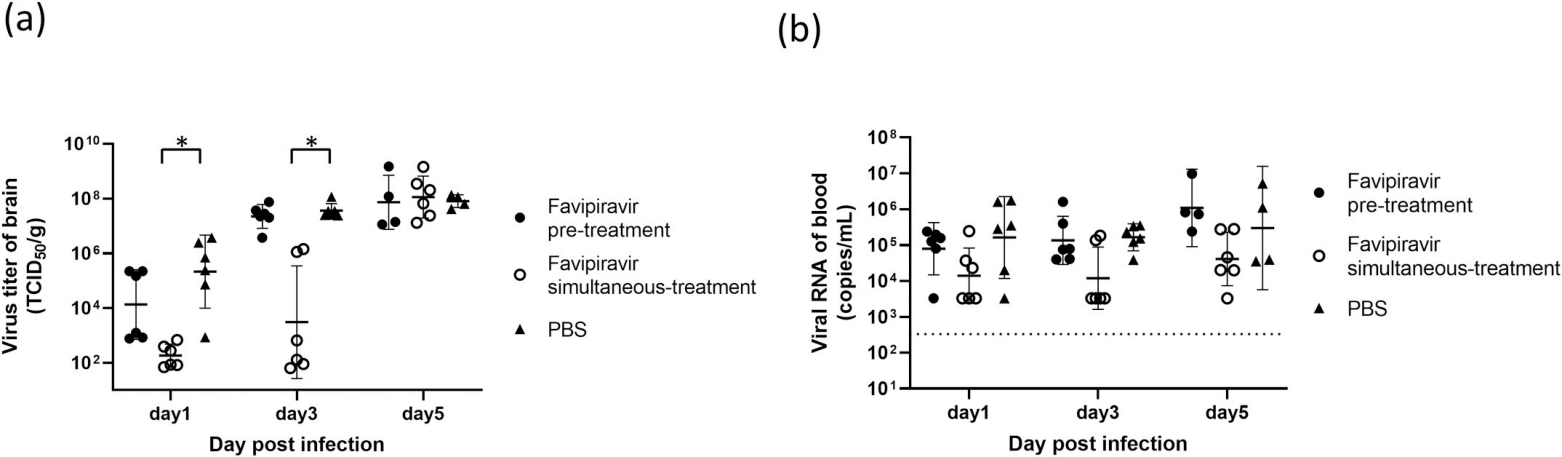
Analysis of the virus titers in the brain and blood viral RNA levels in JCV-infected C57BL/6J mice treated with FPV and PBS. Mice (4–6/group) were inoculated intracerebrally with 1.0×10^4^ TCID_50_ JCV. FPV (300 mg/kg/day) and PBS treatments were initiated 2 days prior to virus inoculation (pre-treatment) or on the same day of virus inoculation (simultaneous-treatment) and continued for 5 days. The virus titers in the brain (a) and the blood RNA levels (b) in JCV-infected C57BL/6J mice, 1, 3, and 5 dpi are shown. The data are obtained from a single experiment. Each bar represents the geometric mean with the geometric standard deviation. The dotted line indicates the limit of detection of RT-qPCR. Asterisks denote significant differences (p < 0.05).

The virus titers in the brain and blood of mice treated with FPV *versus* PBS were monitored, 1, 3, and 5 dpi (Fig 4). In all groups, the virus titers in the brain increased over time and showed peaks at 5 dpi: 7.3×10^7^ TCID_50_/g, 1.1×10^8^ TCID_50_/g, and 8.0×10^7^ TCID_50_/g in FPV pre-treated, FPV simultaneous-treated, and control mice, respectively. At 1 and 3 dpi, the virus titers in the brain were, however, significantly lower in the FPV simultaneous-treated relative to PBS treated animals (p = 0.002, both 1 and 3 dpi). Additionally, at 1 dpi, the virus titers in the brain tended to be lower in FPV pre-treated (1.3×10^4^ TCID_50_/g) relative to PBS treated (2.1×10^5^ TCID_50_/g) animals; however, no significant difference was detected (Fig 4A). Since the viral titers were under the detection level (<10^3^ TCID_50_/mL) in blood samples, the viral RNA levels were investigated. A similar pattern as that of the brain was observed. The values for 1 and 3 dpi were as follows: 7.9×10^4^ copiesmL and 1.4×10^5^ copies/mL in the FPV pre-treatment group, 1.4×10^4^ copies/mL and 1.2×10^4^ copies/mL in the FPV simultaneous-treatment group, and 1.6×10^5^ copies/mL and 1.7×10^5^ copies/mL in the PBS-treated group. Of note, the levels increased at 5 dpi: 1.1×10^6^ copies/mL, 4.1×10^4^ copies/mL, and 3.0×10^5^ copies/mL, respectively (Fig 4B). Importantly, the viral RNA levels in the blood tended to be lower in the FPV simultaneous-treated animals compared with the FPV pre-treated and the PBS-treated animals during the observation period, although not in a significant fashion.

## Discussion

In this study, we show that FPV exhibited an inhibitory effect on JCV replication *in vitro*, as well as a significant therapeutic effect *in vivo*. There have not been any previous studies, to the best of our knowledge, showing the efficacy of FPV against JCV infections.

FPV, RBV, and 2’-FdC are recognized as broad-spectrum inhibitors against several bunyaviruses [19,20,33–35]. Remarkably, all of the drugs showed antiviral activity against JCV *in vitro* (Fig 1). In the context of JCV replication, FPV showed a 6 log_10_ and a 3 log_10_ reduction in Vero and N2A cells with IC_90_ values of 41.0 and 20.7 μM, respectively. Importantly, these values are similar to those reported for other bunyaviruses, including LACV (IC_50_: 32 μM) and RVFV (IC_50_: 32 μM) [39], although such a parallel must be drawn carefully. Of note, the inhibitory effect of 2’-FdC in the context of *in vitro* JCV replication was the highest among the three drugs; RBV showed similar efficacy to that of FPV *in vitro*. For LACV, which belongs to the CSG, together with JCV, the IC_50_ of RBV and 2’-FdC was 60.7 and 2.9 μM in Vero 76 cells, respectively [35]; therefore the results on LACV are mostly consistent with the results obtained in this study for JCV. Considering that FPV exhibited low cytotoxicity against Vero [20,35,39] and N2A cells, we expected that FPV would show a higher efficacy (and better tolerability) in our mouse model.

As the three drugs showed *in vitro* inhibitory effects against JCV on Vero cells, in the next step, their efficacy was evaluated *in vivo*. For this purpose, a JCV infection lethal mouse model was developed. As per previous reports, JCV did not cause neurologic disease in weanling Swiss Webster and C57BL/6 mice after intraperitoneal inoculation due to an inability of the viruses to penetrate the blood-brain barrier or replicate in the periphery [3,40]. Additionally, JCV did not induce neuroinvasive disease in adult or aged mice after intraperitoneal inoculation [40]. Contrastingly, adult C57BL/6 mice showed neurologic disease when 1.0×10^4^ JCV plaque-forming units were inoculated intranasally, possibly because JCV directly reached the central nervous system (CNS) [40]. In this study, to develop an appropriate model for determining the efficacy of different drugs against JCV infection, adult C57BL/6J mice were infected intracerebrally or intranasally. All mice developed neurologic disease when they were challenged intracerebrally with 1.0×10^4^ TCID_50_ JCV. When the same dose (1.0×10^4^ TCID_50_) of viruses was administered via the intranasal route, only a small number of mice showed neurologic disease; of note, these mice did not show any weight loss, indicating that the health status of the mice was adequate. We speculated that mice purchased from different vendors or the minor differences in our protocol led to this discrepancy. Therefore, to evaluate the efficacy of drugs against JCV infection, the intracerebral route of infection, and the dose of 1.0×10^4^ TCID_50_ JCV were selected.

None of the drugs substantially improved the survival rate of infected mice. However, FPV prolonged their survival time for several days in both pre- and simultaneous-treatment groups. The other drugs did not significantly prolong the survival time of animals. In line with our results, it was previously reported that RBV and 2’-FdC were ineffective in treating LACV infection *in vivo* [35]. Additionally, RBV showed no antiviral activity against other orthobunyaviruses such as Oropouche, Caraparu, Guama, Guaroa, or Tacaiuma viruses in the context of a mouse model [41]. A possible explanation for these results is the hypothesis that these drugs probably do not cross the blood-brain barrier efficiently and, therefore do not penetrate well into the CNS [35,42,43]. This said, no pharmacokinetic or drug metabolism studies on 2’-FdC are available to support our hypothesis. RBV was assessed in a clinical trial (phaseI, IIA, and IIB) of children infected with LACV, which was, however, discontinued due to adverse reactions [44]. Nevertheless, RBV is occasionally prescribed to treat JCV infection [45]. However, our results clearly suggest that FPV would be an effective option for treating patients infected with JCV.

To the best of our knowledge, no previous study has reported that FPV is effective against viruses associated with neurologic diseases in animal models infected via the intracerebral route. However, FPV was previously defined as an effective antiviral agent *in vivo*, against various RNA viruses of the *Bunyaviridae* family, including CCHFV [19], Punta Toro virus [39], and SFTSV [20]. Moreover, FPV also demonstrated *in vivo* antiviral efficacy against neurotropic viruses in other families including WNV [21], WEEV [22], and RABV [23]. In these studies, the viruses were inoculated via peripheral routes such as the intramuscular, subcutaneous, and intraperitoneal routes. In the present study, an intracerebral route was used since the mice inoculated with JCV did not show clinical signs when infected through a peripheral route, as described above [40]. The intracerebral inoculation route is thought to be a more challenging way to evaluate drug efficacy because the virus can directly bypass the blood-brain barrier, as compared to peripheral routes. The use of this route may introduce an important disadvantage for evaluating the efficacy of drugs: it does not account for the natural incubation period. However, regardless of this disadvantage, in this study, we showed that FPV delayed the onset of neurologic disease in mice infected intracerebrally with JCV.

Consistent with the survival curves, the virus titers in the brains of mice were clearly lower in FPV simultaneous-treated versus PBS-treated animals at 1 and 3 dpi. However, the virus titers in the brain and blood viral RNA load of mice pre-treated with FPV were not significantly lower than those of PBS-treated animals. Considering that these values were elevated after FPV was discontinued, it is clear that the treatment duration had an impact on the treatment efficacy. In other words, a longer treatment time might further prolong the efficacy; however, it should be ascertained in future studies. A limitation of this study was that we could not evaluate the efficacy of FPV after the onset of neurological symptoms, as this point was defined as the humane endpoint. Therefore, it is still unclear whether FPV is effective in the context of symptomatic mice.

In conclusion, although the intracerebral inoculation route is thought to be a more challenging way to evaluate drug efficacy, FPV showed an inhibitory effect on the replication of JCV *in vitro* and delayed the onset of neurologic diseases in C57BL/6J mice intracerebrally infected with JCV. Most patients infected with JCV present a mild illness, but severe neuroinvasive diseases are reported in some cases of JCV infections. Therefore, this study provides a basis for the development of a specific antiviral treatment for patients infected with JCV and establishment of an effective animal model for JCV infections.

## Supporting information

S1 Fig Survival of JCV-infected C57BL/6J mice treated with FPV, RBV, and 2’-FdC in a single independent experiment. Five mice in each group were inoculated intracerebrally with 1.0×10^4^ TCID_50_ JCV. Treatments were commenced (a, b) 2 days prior to viral inoculation (pre-treatment) or (c, d) on the same day of viral inoculation (simultaneous-treatment) and continued for 5 days. The mice were treated intraperitoneally with FPV (300 mg/kg/day), RBV (100 mg/kg/day), 2’-FdC (100 mg/kg/day), or PBS as negative control once a day. Survival was determined using the Kaplan-Meier analysis. The data of the single independent experiments are shown in each figure.
(TIF)

S1 Table Median survival time (day) of JCV-infected C57BL/6J mice treated with FPV, RBV, 2’-Fdc, and PBS in a single independent experiment. P-value was calculated using the log-rank test.
(XLSX)
