# Supplementary figures and images for: Favipiravir treatment prolongs the survival in a lethal mouse model intracerebrally inoculated with Jamestown Canyon virus
Source: PLoS Negl Trop Dis. 2021 Jul 2;15(7):e0009553. doi: 10.1371/journal.pntd.0009553 (PMC8281987; doi:10.1371/journal.pntd.0009553)

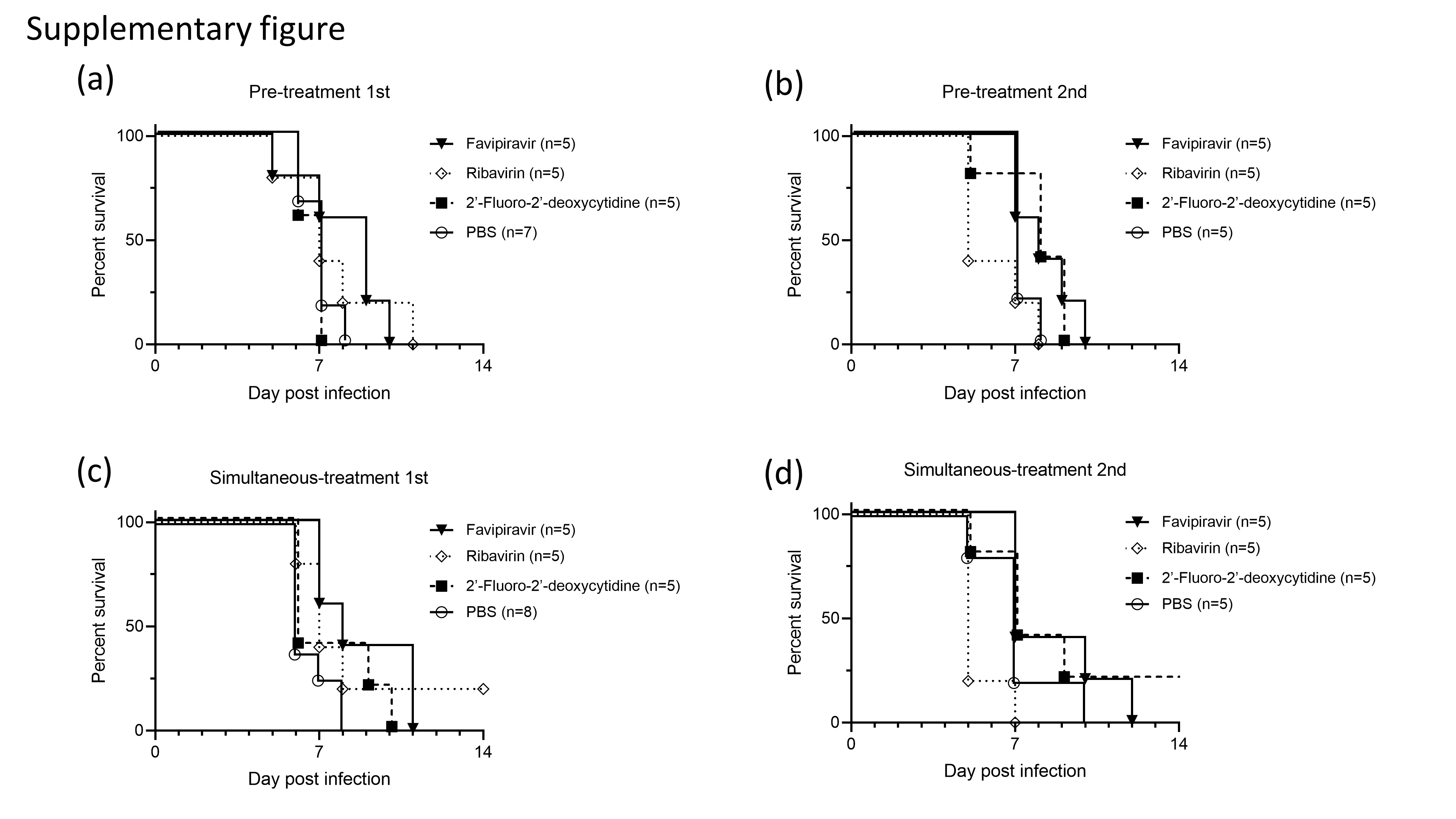

Supplement: S1 Fig — Five mice in each group were inoculated intracerebrally with 1.0×10^4^ TCID50 JCV. Treatments were commenced (a, b) 2 days prior to viral inoculation (pre-treatment) or (c, d) on the same day of viral inoculation (simultaneous-treatment) and continued for 5 days. The mice were treated intraperitoneally with FPV (300 mg/kg/day), RBV (100 mg/kg/day), 2’-FdC (100 mg/kg/day), or PBS as negative control once a day. Survival was determined using the Kaplan-Meier analysis. The data of the single independent experiments are shown in each figure. (TIF) [file pntd.0009553.s001.TIF]
